# Paranasal Sinus CT and Polysomnographic Findings in Adults with Cystic Fibrosis: Implications for Obstructive Sleep Apnea

**DOI:** 10.3390/pathophysiology33010006

**Published:** 2026-01-14

**Authors:** Matthias Welsner, Sarah Dietz-Terjung, Svenja Strassburg, Dirk Westhölter, Sivagurunathan Sutharsan, Christoph Schöbel, Christian Taube, Florian Stehling, Cornelius Kürten, Cornelius Deuschl, Michael Forsting, Sebastian Zensen, Johannes Haubold, Benedikt M. Schaarschmidt, Marcel Opitz

**Affiliations:** 1 Department of Pulmonary Medicine, University Hospital Essen—Ruhrlandklinik, Adult Cystic Fibrosis Center, University of Duisburg-Essen, 45239 Essen, Germany; 2 Department of Sleep and Telemedicine, University Hospital Essen—Ruhrlandklinik, University of Duisburg-Essen, 45141 Essen, Germany; 3 Pediatric Pulmonology and Sleep Medicine, Cystic Fibrosis Center, Children’s Hospital, University of Duisburg-Essen, 45141 Essen, Germany; 4 Department of Otorhinolaryngology, Head and Neck Surgery, University Hospital Essen, University of Duisburg-Essen, 45141 Essen, Germany; 5 Institute of Diagnostic and Interventional Radiology and Neuroradiology, University Hospital Essen, University of Duisburg-Essen, 45141 Essen, Germany

**Keywords:** cystic fibrosis, adults, polysomnography, apnea-hypopnea index, obstructive sleep apnea, sinus computed tomography, chronic rhinosinusitis, Lund-Mackay score

## Abstract

Objective: To assess whether chronic rhinosinusitis (CRS) severity is associated with obstructive sleep apnea (OSA) in adult people with cystic fibrosis (pwCF). Methods: We conducted a retrospective single-center study of 44 adults with CF who underwent overnight polysomnography (PSG), Epworth Sleepiness Scale (ESS) assessment, and sinus computed tomography (CT). CRS severity was quantified using the Lund–Mackay score (LMS) and the main nasal cavity score (MNCS). OSA was defined by Apnea–Hypopnea Index (AHI) thresholds per American Academy of Sleep Medicine criteria. Results: Participants had a mean age of 31.1 ± 8.4 years and a mean percent predicted FEV1 of 51.8 ± 15.7. Sinus CT showed radiological evidence of CRS in all participants. Mean AHI was 5.3 ± 4.4/h; 48% had AHI ≥ 5/h. There were no significant differences between pwCF with and without OSA in age, sex, BMI, lung function, total sleep time, sleep efficiency, or ESS score (all *p* > 0.05). Mean LMS and MNCS did not differ between OSA and non-OSA groups (both *p* > 0.05), and neither score correlated with PSG parameters or ESS (all *p* > 0.05). Receiver operating characteristic (ROC) analysis demonstrated low discriminative ability of LMS and MNCS for predicting OSA (AUCs < 0.70, *p* < 0.05). Conclusions: In this cohort of adults with CF, CT-based CRS severity was not associated with OSA. Given the substantial prevalence of OSA observed, PSG screening should be considered irrespective of CRS severity.

## 1. Introduction

Cystic fibrosis (CF) is a monogenetic autosomal recessive disease caused by mutations in the Cystic Fibrosis transmembrane conductance regulator (CFTR) gene [1]. This results in impaired anion transport across epithelial cell membranes, leading to the production of abnormally thick secretions in exocrine glands. In the respiratory tract, this viscous mucus impairs mucociliary clearance, causing ciliary dyskinesia, chronic inflammation, and recurrent infections, which contribute to progressive lung disease and chronic rhinosinusitis (CRS) [2]. According to the 2020 European Position Paper on Rhinosinusitis and Nasal Polyps (EPOS), CRS is categorized into two principal subtypes: primary (idiopathic) and secondary, which arises in the context of systemic diseases, immunodeficiencies, or ciliary dyskinesia [3]. In the case of CF, CRS is classified as secondary, with its pathogenesis directly attributable to mutations in the CFTR gene Therefore, CRS, with or without nasal polyps, is a frequent comorbidity affecting both pediatric and adult people with cystic fibrosis (pwCF). Sinus computed tomography (CT) reveals mucosal thickening in at least 90% of pwCF, although only a minority report associated symptoms [4,5,6]. Common symptoms of CF-CRS, often starting early in life, include reduced or absent sense of smell (hyposmia/anosmia), chronic nasal congestion and obstruction, persistent rhinorrhea, and facial pain [5]. CRS in CF can negatively affect quality of life and potentially trigger pulmonary exacerbations due to the presence of pathogens, such as *Pseudomonas aeruginosa*, within the sinuses [2,5,7].

Sleep disturbances and sleep-disordered breathing (SDB), including obstructive sleep apnea (OSA), nocturnal hypoxemia, and nocturnal hypoventilation, are recognized as significant comorbidities in adult pwCF [8,9,10,11,12]. These conditions negatively impact the physical and mental well-being of pwCF, potentially contributing to reduced health-related quality of life (HrQoL), excessive daytime sleepiness (EDS), disrupted circadian rhythms, increased restless legs syndrome symptoms, insomnia, and decreased physical activity [13,14,15,16,17,18]. Despite their prevalence and potential consequences, routine screening for sleep disturbances and SDB is not yet standard practice in most cystic fibrosis centers [19].

Research investigating SDB in adult pwCF has largely focused on lower respiratory tract physiology. While nocturnal hypoxemia and hypoventilation correlate with the severity of underlying lung disease [20,21], no consistent association has been identified between the presence or severity of OSA and the degree of lung disease impairment [12,22,23].

The prevalence of OSA in the general population is substantial, affecting up to 24% of middle-aged men and 9% of middle-aged women [24]. In contrast, OSA is significantly more prevalent in pwCF, affecting up to 70% of children and 40% of adults [12,25]. Notably, children and adults with CF exhibit a threefold increased risk of moderate-to-severe OSA compared to matched non-CF controls (age, sex, race, BMI) [10].

Despite the high prevalence of OSA in pwCF, particularly in children, the underlying mechanisms and the specific role of upper respiratory tract abnormalities remain incompletely understood. While some studies suggest a link between structural changes like enlarged tonsils, pharyngeal pillars, and CRS and OSA in pediatric pwCF [26], others have found no correlation between CT findings of sinus disease and OSA [22]. Furthermore, the relationship between CF-related CRS and OSA specifically in adult pwCF has not yet been investigated.

Obesity, male sex, and advancing age are established determinants of obstructive sleep apnea (OSA) risk in the general population. In contrast, similar to findings in people with cystic fibrosis (pwCF), evidence on the contribution of upper airway structural and inflammatory alterations to OSA occurrence is heterogeneous. Rhinosinusitis has been reported to be significantly associated with increased OSA severity, with an effect size comparable to that of obesity [27], and one study found that nearly 65% of patients with chronic rhinosinusitis (CRS) also had OSA [28]. Conversely, Alt et al. identified comorbid CRS in only 15% of OSA patients, and Mahdavinia et al. observed no differences in polysomnographic outcomes between adult OSA patients with and without CRS [29,30].

This study aimed to investigate the association between the radiographic severity of CF-CRS, as assessed by sinus CT, and the presence and severity of OSA in adult patients with cystic fibrosis, to guide clinical management.

## 2. Materials and Methods

### 2.1. Study Design and Participants

This study was designed as a follow-up to previous research examining SDB in adult pwCF [12]. The study employed a retrospective, single-center design and was conducted at the adult CF center of the Ruhrlandklinik Essen, Germany. The source population consisted of all pwCF aged 18 years and older who had been referred to the institutional sleep laboratory for diagnostic evaluation of SDB, including suspected SDB, between 1 September and 31 December 2020. To be eligible for inclusion, patients were also required to have undergone paranasal sinus CT as part of routine clinical management within the same period. These imaging procedures were performed in accordance with standardized departmental protocols for sinus CT evaluation.

The study protocol was reviewed and approved by the local ethics committee of the University Hospital Essen (approval numbers 19-8961-BO and 19-8797-BO). All procedures were conducted in accordance with the ethical principles stated in the Declaration of Helsinki regarding research involving human subjects. Prior to participation, each patient was provided with detailed written and verbal information explaining the study objectives, procedures, and any potential risks. Written informed consent was obtained from all participants before inclusion in the database. Clinical and diagnostic data were subsequently extracted from patient records and analyzed in accordance with institutional data protection regulations.

Anthropometric and pulmonary function assessments were performed either on the same day as the diagnostic polysomnography or on the following day. Body mass index (BMI) was calculated as body weight (kg) divided by height squared (m^2^). Pulmonary function testing was conducted using a calibrated JAEGER MasterScreen Body plethysmograph (CareFusion, Höchberg, Germany) according to the standardized procedures outlined by the American Thoracic Society (ATS) guidelines [31]. The spirometric parameters recorded included forced vital capacity (FVC), forced expiratory volume in one second (FEV_1_), and forced expiratory flow between 25% and 75% of the vital capacity (FEF_25-75_). All measurements were expressed as both absolute values and percent predicted values, calculated using the Global Lung Function Initiative (GLI) reference equations [32].

### 2.2. Polysomnography and the Epworth Sleepiness Scale

In-laboratory overnight polysomnography (PSG) was conducted in accordance with established protocols previously detailed elsewhere [12]. For each patient, the diagnostic recording included two electroencephalogram (EEG) channels, two electrooculogram (EOG) channels, and electromyographic (EMG) recordings from the submental and bilateral tibialis muscles. Respiratory effort was assessed via rib cage and abdominal inductance pneumography, while oxygen saturation was continuously monitored using pulse oximetry. Nasal airflow was measured with a nasal cannula connected to a pressure transducer, sampled at a frequency of 20 Hz. Data acquisition was performed using a digital polygraph system (Nox Medical, Reykjavík, Iceland). Scoring of sleep stages and respiratory events was performed in accordance with the American Academy of Sleep Medicine (AASM) criteria, with apnea and hypopnea events defined according to current AASM standards [33]. Significant nocturnal oxygen desaturation was defined as a peripheral oxygen saturation (SpO_2_) of less than 90% for ≥5% of the total sleep time (TST), combined with a minimum nadir SpO_2_ of ≤85% [34]. To minimize inter-rater variability, all polysomnographic data were acquired according to standardized laboratory procedures and subsequently scored in a blinded fashion by a senior investigator certified by the German Sleep Society (DGSM). The scorer was not involved in patient care and had no prior knowledge of patient clinical characteristics or other study data. The apnea–hypopnea index (AHI) was calculated as the total number of apneas and hypopneas per hour of sleep. In addition to the overall AHI, two sub-indices were determined: AHI during rapid eye movement (AHI_REM_) sleep and AHI during non-rapid eye movement (AHI_NREM_) sleep, which distinguish respiratory events occurring in different sleep stages. OSA was diagnosed when the AHI was ≥5 events/hour.

Daytime sleepiness was evaluated using the Epworth Sleepiness Scale (ESS), a validated self-administered questionnaire that assesses the likelihood of dozing off in eight common daily situations. Each item is scored from 0 (“would never doze”) to 3 (“high chance of dozing”), yielding a possible total score between 0 and 24. Scores greater than 10 are indicative of excessive daytime sleepiness. The ESS was completed by participants on the morning following the diagnostic PSG [35].

### 2.3. Computed Tomography (CT) Acquisition

A non-contrast-enhanced computed tomography (CT) scan was performed using a 64-detector row, single-source scanner (SOMATOM Definition AS, Siemens Healthineers, Erlangen, Germany) to evaluate the paranasal sinuses and nasal cavity. Patients were scanned in the supine position. The scan field of view (FOV) extended from the frontal sinuses to the nasopharynx but excluded the oropharynx and hypopharynx intentionally in order to reduce radiation exposure while maintaining diagnostic quality for assessing the sinuses and nasal cavities. The scanning parameters were as follows: gantry rotation time of 1.0 s, collimation of 64 × 0.6 mm, tube current–time product of 50 mAs and tube voltage of 120 kV. Image reconstruction was performed using high-resolution convolution kernels (H60S and J30S). All datasets were reconstructed in the bone window with a slice thickness of 1.0 mm, generating both axial and coronal imaging planes. To minimise radiation exposure, automatic tube current modulation (CARE Dose4D) and automatic tube voltage selection (CARE kV algorithm) were applied, as supplied by the manufacturer (Siemens Healthineers, Erlangen, Germany).

### 2.4. Lund–Mackay Score (LMS) and Main Nasal Cavity Score (MNCS)

Paranasal sinus pathology was quantified using the Lund–Mackay Score (LMS), a validated and widely implemented radiological grading system for sinus disease [36]. In this scoring method, individual sinus regions are assigned numerical values based on the degree of mucosal thickening and luminal obstruction visualized on CT scans. The mucosal status of each evaluated region is graded as follows: 0 = no mucosal swelling, 1 = partial opacification/mild mucosal swelling, and 2 = complete opacification/total mucosal obstruction. The LMS assesses the following regions bilaterally: frontal sinus, maxillary sinus, sphenoid sinus, anterior ethmoidal sinus, and posterior ethmoidal sinus. In addition, the ostiomeatal complex is scored separately for each side, with 0 points indicating no mucosal swelling and 2 points indicating ostiomeatal unit involvement.

The LMS yields a minimum possible score of 0 and a maximum of 12 points per side, resulting in a total score range of 0–24 for both sides combined. In the present study, an extended scoring protocol was applied to enhance evaluation of the nasal cavity. This extension—designated as the Main Nasal Cavity Score (MNCS)—was determined bilaterally after the LMS scoring and graded using the same three-level scale (0 = no mucosal swelling, 1 = mild mucosal swelling, 2 = complete mucosal obstruction).

**Figure 1:**
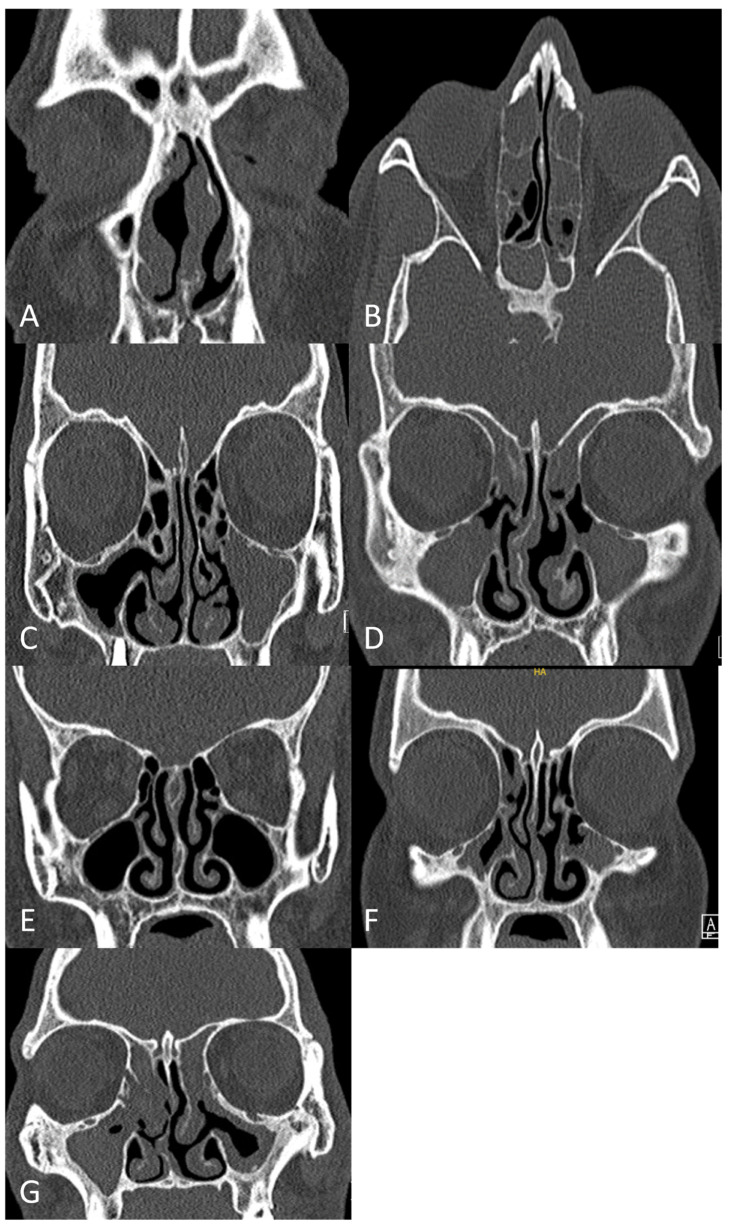
Representative non-contrast-enhanced sinus computed tomography (CT) images illustrating the application of the Lund–Mackay Score (LMS) for radiological grading of paranasal sinus disease, along with the extended evaluation of the main nasal cavity using the Main Nasal Cavity Score (MNCS). (**A**) Right frontal sinus: LMS = 1; left frontal sinus: LMS = 2. (**B**) Right anterior ethmoidal sinus: LMS = 2; left anterior ethmoidal sinus: LMS = 2; right posterior ethmoidal sinus: LMS = 1; left posterior ethmoidal sinus: LMS = 1; right sphenoidal sinus: LMS = 2; left sphenoidal sinus: LMS = 2. (**C**) Right maxillary sinus: LMS = 1; left maxillary sinus: LMS = 2. (**D**) Right osteomeatal complex: LMS = 2; left osteomeatal complex: LMS = 2. (**E**) Right main nasal cavity: MNCS = 0; left main nasal cavity: MNCS = 0. (**F**) Right main nasal cavity: MNCS = 1; left main nasal cavity: MNCS = 0. (**G**) Right main nasal cavity: MNCS = 2; left main nasal cavity: MNCS = 0.

Sinus CT scans were independently reviewed and scored by two board-certified radiologists, blinded to other patient data. The mean values of the independently assigned LMS and MNCSs were calculated and used for all subsequent statistical analyses (Figure 1).

### 2.5. Statistical Analysis

All statistical analyses were conducted using IBM SPSS Statistics software, version 29.0 (SPSS Inc., Chicago, IL, USA). Data are presented as mean ± standard deviation (SD) unless otherwise indicated. The Shapiro–Wilk test was applied to evaluate the normality of data distribution. For comparisons between two independent groups, Student’s *t*-test was used for normally distributed variables, whereas the Mann–Whitney U test was employed for non-normally distributed data. Differences between categorical variables were assessed using the chi-square (χ^2^) test.

Linear associations between continuous variables were examined using Pearson’s correlation coefficient (r). Inter-rater reliability was assessed using the intraclass correlation coefficient (ICC) to evaluate the consistency of ratings across independent observers. An ICC value ≥ 0.75 was considered to indicate excellent reliability, 0.60–0.74 moderate to good reliability, and <0.60 poor reliability. Receiver operating characteristic (ROC) curve analysis was performed to identify predictors of OSA, using the LMS, the MNCS, and the combination of both as independent variables. ROC curve plotting and calculation of test parameters, including area under the curve (AUC), sensitivity, and specificity, were performed in GraphPad Prism, version 10.2.1 (GraphPad Software, San Diego, CA, USA). Statistical significance was defined as a two-tailed *p* value < 0.05.

## 3. Results

### 3.1. Study Population

During the study period, 64 persons with cystic fibrosis (pwCF) underwent in-laboratory overnight polysomnography (PSG) in our sleep facility. Twenty participants without available paranasal sinus CT data were excluded, resulting in a final analytical cohort of 44 pwCF. The mean age was 31.1 ± 8.4 years, with a mean BMI of 21.6 ± 3.5 kg/m^2^ and a mean FEV_1_ of 51.8 ± 15.7% (Table 1).

None of the participants were receiving nocturnal oxygen supplementation, continuous positive airway pressure (CPAP), or bilevel positive airway pressure (BiPAP) therapy. The majority (73%) were homozygous for the F508del CFTR mutation, and 70% were on dual CFTR modulator therapy (tezacaftor/ivacaftor or lumacaftor/ivacaftor). A history of at least one sinus surgery for symptomatic chronic rhinosinusitis was reported by 28 patients (64%).

### 3.2. Sleep Recording

Across the study cohort, the mean total sleep time (TST) was 303.9 ± 38.0 min, with a mean sleep efficiency (SE) of 76.0 ± 9.3% (Table 2). The mean apnea–hypopnea index (AHI) was 5.3 ± 4.4 events/hour, which slightly exceeded the normal range, and respiratory events were predominantly associated with rapid eye movement (REM) sleep (Table 2). The mean ESS score was 6.3 ± 3.8, and 14% of participants had ESS scores > 10, consistent with EDS.

### 3.3. OSA vs. Non-OSA Group Comparisons

Polysomnographic criteria for OSA (AHI ≥ 5 events/hour) were met by 21 of the 44 pwCF (48%). No significant differences were observed between pwCF with and without OSA for age, sex, lung function, BMI, TST, SE, or ESS score (all *p* > 0.05; Table 3). Only 2 of 21 pwCF with OSA (10%) had ESS scores > 10.

Compared with the non-OSA group, pwCF with OSA exhibited significantly lower mean nocturnal oxygen saturation (*p* = 0.002), a higher periodic limb movement index (*p* = 0.013), and a greater likelihood of snoring during sleep (*p* = 0.049) (Table 3).

### 3.4. Association Between CF-CRS and Sleep Parameters

The intraclass correlation coefficient (ICC), demonstrates an excellent agreement between raters with an ICC of 0.82 (95% CI: 0.69–0.90, *p* < 0.001) for the LMS and 0.91 (95% CI: 0.85–0.95, *p* < 0.001) for the MNCS. Sinus CT analysis (Table 2) showed radiological evidence of chronic rhinosinusitis associated with CF-CRS in all participants. The mean total LMS was 14.6 ± 5.2 (range 2–24). Opacification of the main nasal cavity (MNCS ≥ 1) was present in 39 pwCF (89%), with a mean MNCS of 1.0 ± 0.6 (range 0–3). No significant differences in LMS, MNCS, or combined sinus scores were found between the OSA and non-OSA groups (all *p* > 0.05; Table 3). Furthermore, there were no significant correlations between AHI (overall, REM-specific, or NREM-specific), ESS score, and any sinus CT–derived variable (all *p* > 0.05; Table 4).

**Figure 2:**
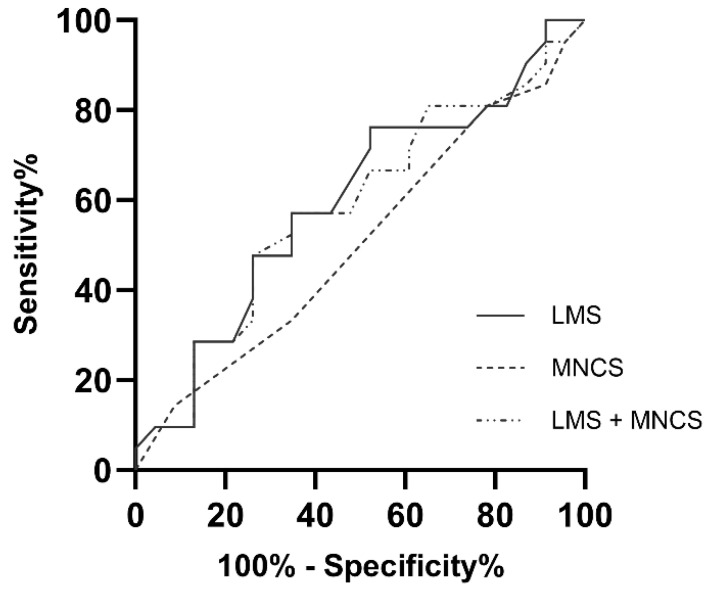
Receiver operating characteristic (ROC) curves for the Lund–Mackay Score (LMS), Main Nasal Cavity Score (MNCS), and the combined LMS + MNCS to predict obstructive sleep apnea (OSA) in adult people with cystic fibrosis (pwCF). The curves illustrate the discriminatory performance of each CT-derived score, with corresponding area under the curve (AUC) values, sensitivity, and specificity reported in the text.

Receiver operating characteristic (ROC) analysis (Figure 2) indicated poor discriminatory performance of all sinus CT parameters for predicting OSA in adult pwCF. The highest area under the curve (AUC) was observed for LMS (AUC = 0.597; *p* = 0.269), while MNCS yielded an AUC of 0.507 (*p* = 0.934). Combining LMS and MNCS did not improve predictive accuracy (AUC = 0.586; *p* = 0.330). Overall, the sensitivity and specificity values for these CT-derived scores were low.

### 3.5. Differences in Clinical Characteristics and Polysomnographic Findings in Relation to CFTR Modulator Use

No significant differences were observed between CF modulator-treated and non-treated adult pwCF in age, sex, or BMI (all *p* > 0.05). Pulmonary function, as assessed by ppFEV_1_, was significantly lower in the modulator-treated group (*p* = 0.043). Total sleep time (*p* = 0.088) and sleep efficiency (*p* = 0.175) were higher in the modulator group, though not statistically significant. The AHI and ODI were slightly higher in the treated group but did not reach statistical significance (both *p* > 0.05). ESS scores were comparable between groups (*p* = 0.522). No significant differences were found in sinus CT parameters (all *p* > 0.05). For a complete overview, see Table 5.

## 4. Discussion

This study represents a systematic sleep investigation in adult pwCF to examine the relationship between CF-CRS and OSA, using sinus CT for objective quantification of sinonasal disease. Employing standardized radiological scoring systems, including the Lund–Mackay Score (LMS) and the Main Nasal Cavity Score (MNCS), alongside comprehensive in-laboratory polysomnography, we assessed both structural sinonasal pathology and sleep-disordered breathing. The principal finding of this work is that neither the presence nor the radiological severity of CF-CRS, as determined by sinus CT scores, was significantly associated with the occurrence of OSA in this cohort.

This lack of correlation persisted across multiple sleep parameters, including overall apnea–hypopnea index (AHI), stage-specific indices (AHI_REM_ and AHI_NREM_), and subjective daytime sleepiness scores (ESS). Furthermore, receiver operating characteristic (ROC) analyses demonstrated poor discriminatory performance of all CT-derived measures for identifying patients with OSA, suggesting that sinus imaging in pwCF does not provide reliable predictive value for OSA.

Unlike pediatric and adolescent pwCF, data on the coexistence of OSA in adults with CF are limited. A meta-analysis by Sousa et al. reported a pooled prevalence of OSA as high as 65% among children and adolescents with CF, with no identified correlation between standard clinical variables and OSA occurrence [37]. In contrast, Perin et al., examining an adult cohort of 51 pwCF, found that only two patients (3.9%) met diagnostic criteria for OSA; notably, both individuals had comorbid CF-CRS, EDS, and snoring [20]. More recently, Shakkottai et al. documented a higher prevalence of OSA (53%) in a mixed-age pwCF cohort [23]. The Shakkottai study employed clinical examination rather than CT to evaluate the upper airway, yet yielded similar findings regarding the absence of correlation between nasal cavity obstruction and OSA. In the current study, we likewise found no significant relationship between CT-derived measures of main nasal cavity opacification and OSA presence. Taken together, these results are consistent with prior observations in both adult pwCF and individuals without CF, suggesting that nasal obstruction—whether clinically or radiologically determined—is not a primary pathophysiological driver of OSA [23,38].

In populations without CF, several studies have demonstrated a positive association between higher LMS values and increased severity of OSA [27]. In contrast, the present findings indicate that in adult pwCF, the LMS has limited predictive utility for assessing CF-CRS in relation to OSA risk. Specifically, LMS was not suitable as a discriminative tool for identifying pwCF with OSA in this cohort. This interpretation is supported by the work of Casserly et al., who found no significant differences in LMS between pwCF with and without clinical CRS symptoms, suggesting the low impact of LMS values on symptom expression in this population [6]. Similarly, Eischen et al. compared the well-established Sinonasal Outcome Test-22 (SNOT-22) with LMS and reported poor agreement between the two measures; notably, LMS did not accurately reflect the patients’ reported sinonasal symptom burden [39]. To date, no study has specifically explored the impact of symptomatic versus asymptomatic CF-CRS on the prevalence or severity of OSA in pwCF. Interestingly, in the general population, the SNOT-22 has demonstrated usefulness as a screening tool for identifying patients with OSA and coexisting CRS [40]. Whether patient-reported sinonasal outcome measures, such as the SNOT-22, could similarly improve OSA detection or risk stratification in adult pwCF remains an open question and warrants targeted investigation in future studies.

Research into the pathogenesis of OSA has highlighted its multifactorial nature, involving a complex interplay of anatomical, neuromuscular, and physiological factors. The extent to which these established mechanisms can be extrapolated to pwCF remains uncertain. Classical risk factors for OSA—such as older age, male sex, and obesity—are generally uncommon in pwCF. Instead, other pathophysiological contributors may be more relevant in this population. These include structural and mechanical alterations in the upper airway resulting from chronic infection, inflammation, or previous surgical interventions; impaired neuromuscular control of the pharyngeal airway; and changes in lung volume that may affect pharyngeal patency through modifications in upper airway pressure dynamics. Such CF-specific anatomical and physiological characteristics may influence OSA risk and phenotype in ways distinct from the general population, potentially explaining the lack of correlation between sinonasal disease severity and OSA observed in this study [41,42].

Emerging evidence indicates that both systemic and upper airway inflammatory processes may play a substantial role in the pathogenesis of OSA [43,44]. In pwCF, chronic bronchial neutrophilic airway inflammation is a hallmark of disease, accompanied by excessive production of reactive oxygen species and release of proteolytic enzymes [45]. Comparable inflammatory patterns have been documented in the upper airway mucosa of pwCF, reflecting a widespread inflammatory milieu that extends beyond the lower respiratory tract [46]. Persistent inflammation within the airways can lead to structural remodeling, mucosal thickening, and impaired neuromuscular function, collectively contributing to airway dysfunction [47,48,49]. In addition, the presence of OSA and its characteristic chronic intermittent hypoxia may further perpetuate and amplify inflammatory processes in the upper airway through oxidative stress, endothelial activation, and recruitment of inflammatory mediators. This bidirectional relationship between inflammation and OSA could theoretically influence upper airway collapsibility and contribute to disease severity, although the specific mechanisms and clinical impact in pwCF remain to be fully elucidated [50,51].

Defective CFTR function appears to influence respiratory muscle physiology [52,53]. CFTR is expressed in both smooth and striated muscle cells within the respiratory system [54,55], and its dysfunction can disrupt calcium homeostasis in the sarcoplasmic reticulum, ultimately impairing the contractile properties of muscle fibers. In airway smooth muscle, CFTR defects have been linked to increased bronchial hyperreactivity and altered cell proliferation, changes that resemble pathophysiological features observed in bronchial asthma [52]. Given the well-documented overlap between chronic obstructive pulmonary disease (COPD) and asthma with OSA in the general population [56], it is plausible that airway muscle dysfunction secondary to CFTR-related abnormalities may contribute to airway instability during sleep in pwCF. This mechanism could help explain the relatively high prevalence of OSA reported in some adult pwCF cohorts, despite the absence of classical OSA risk factors such as obesity and older age. Further research is warranted to elucidate the extent to which CFTR-mediated changes in airway muscle function directly influence OSA pathogenesis in this population.

In our study cohort, treatment with the triple CFTR modulator therapy elexacaftor/tezacaftor/ivacaftor (ETI) was associated with a substantial reduction in the AHI [57]. The precise mechanisms underlying this improvement in SDB remain incompletely understood. In addition, ETI use has been reported to yield sustained improvements in radiological measures of sinonasal disease, as reflected by reduced LMS and improved patient-reported outcomes the SNOT-22 [58]. CFTR modulators function by partially restoring the defective CFTR protein, thereby normalizing dysregulated ion transport across epithelial surfaces. While this molecular correction plausibly impacts multiple pathophysiological pathways, the manner in which ETI influences the complex relationship between sleep parameters, sinus imaging findings, and CF-CRS symptoms remains to be clarified. Theoretically, correction of CFTR channel dysfunction may lead to the normalization of various physiological processes, including attenuation of airway inflammation, improvement of mucus rheology, reduction of pulmonary obstruction, and mitigation of hyperinflation in both the upper and lower airways. These changes could, in turn, enhance pharyngeal airway stability during sleep and reduce OSA severity. Support for this hypothesis comes from recent experimental data demonstrating that CFTR modulators decrease airway smooth muscle cell proliferation and restore sarcoplasmic reticulum calcium reuptake kinetics, thereby potentially improving respiratory muscle and upper airway function [52].

OSA is a common sleep-related breathing disorder that has been linked to numerous adverse health outcomes, including increased risk of arterial hypertension, atrial fibrillation, heart failure, coronary artery disease, pulmonary hypertension, stroke, and even all-cause mortality [59]. Given the substantial improvements in CF care in recent decades—particularly with the introduction of CFTR modulators—the life expectancy of pwCF has risen considerably. As pwCF are now living longer, the prevention, early detection, and effective management of comorbidities such as OSA becomes increasingly important to avoid long-term complications of untreated disease. Recent observations indicate that the prevalence of OSA among adults with CF may be higher than previously appreciated, with some studies reporting rates exceeding 50% in certain cohorts [23]. Importantly, no reliable clinical or radiological predictors have been identified to date for OSA risk in adult pwCF, underscoring the limitations of currently available screening tools. In light of these findings—and given the potentially severe cardiovascular and pulmonary consequences associated with undiagnosed OSA—routine and systematic OSA screening should be considered an integral part of the clinical evaluation of adult pwCF. This approach could facilitate timely diagnosis and appropriate intervention, thereby reducing the risk of late complications and improving overall quality of life in this growing population.

This study has several limitations that warrant consideration. First, the retrospective, single-centre study design has inherent limitations, and the modest sample size introduces potential biases and limits the broader applicability of the observed findings. Consequently, the lack of statistically significant associations should be interpreted cautiously, as the study may have lacked the power to detect clinically relevant effects. Second, no validated questionnaire was administered to distinguish between symptomatic and asymptomatic CF-CRS, nor to explore potential associations between symptom burden and the presence or severity of OSA. This prevents evaluation of the relationship between symptom severity and OSA, which may be more clinically relevant than radiological scores alone. Third, the scoring system employed for the evaluation of the main nasal cavity (MNCS) has not yet undergone formal validation, which may affect its reproducibility and comparability across studies. Finally, neither a clinical inspection nor imaging of the oropharyngeal region was performed. In order to minimise radiation exposure to patients during sinus CT scanning, the oropharyngeal region was excluded from the imaging field of view. This precluded any morphological assessment of this anatomical area in relation to sleep-disordered breathing.

## 5. Conclusions

In summary, our data indicate that the radiological severity of CF-CRS, as assessed by sinus CT, is not associated with OSA in adult pwCF. These findings suggest that factors other than CF-CRS are likely to play a greater role in the development of OSA in this population. Consequently, the presence, absence, or degree of CF-CRS should not be used as a surrogate for OSA risk assessment in adult pwCF.

Given the potential cardiovascular, pulmonary, and neurocognitive consequences of undiagnosed OSA, routine screening should be implemented as part of standard clinical care for adults with CF, independent of sinonasal disease status. Early detection could facilitate timely treatment initiation, thereby mitigating the adverse health outcomes associated with untreated OSA and improving long-term quality of life in this growing patient population.

## Figures and Tables

**Table 1:** Participant demographic and clinical characteristics.

Characteristics	Participants (n = 44)
Age, years	31.1 ± 8.4 (20–49)
Female sex, n (%)	14 (32)
Genotype, n (%)	
F508del homozygous	32 (73)
F508del heterozygous	12 (27)
CFTR modulator therapy, n (%)	
None	13 (30)
Tezacaftor/ivacaftor	29 (66)
Lumacaftor/ivacaftor	2 (4)
Body mass index, kg/m^2^	21.6 ± 3.5 (15.6–31.2)
FEV_1_, L	2.1 ± 0.8 (1.0–4.8)
FEV_1_, % predicted	51.8 ± 15.7 (29.0–96.0)
FVC, L	3.4 ± 1.1 (1.5–6.2)
FVC, % predicted	69.2 ± 18.3 (37.0–105.0)
FEF_25-75_, L/s	1.0 ± 0.7 (0.2–3.9)
FEF_25-75_, % predicted	23.6 ± 14. (5.0–72.0)
Pancreatic insufficiency, n (%)	43 (98)
*Pseudomonas aeruginosa* positive, n (%)	26 (59)
Cystic fibrosis-related diabetes, n (%)	9 (20)
Oxygen supplementation, n (%)	0 (0)
Nocturnal positive pressure ventilation, n (%)	0 (0)
Previous sinus surgery, n (%)	28 (64)

Values are expressed as mean ± standard deviation (range) or as number of patients (percentage), unless otherwise specified. CFTR, cystic fibrosis transmembrane conductance regulator; FEV_1_, forced expiratory volume in one second; FVC, forced vital capacity; FEF, forced expiratory flow.

**Table 2:** Sinus computed tomography (CT) scores and sleep parameters in the total study population of adult people with cystic fibrosis (pwCF).

Parameter	Participants (n = 44)
Lund–Mackay score (LMS)	14.6 ± 5.2 (1.0–24.0)
Main nasal cavity score (MNCS)	1.0 ± 0.6 (0–2.5)
LMS + MNCS	15.5 ± 5.5 (1.5–23.5)
TST, min	303.9 ± 38.0 (218.6–400.0)
Sleep efficiency, %	76.0 ± 9.3 (59.1–93.9)
AHI, events/h	5.3 ± 4.4 (0.0–15.5)
AHI ≥ 5 events/h, n (%)	21 (48)
AHI REM, events/h	12.7 ± 12.5 (0.0–46.2)
AHI NREM, events/h	3.7 ± 3.8 (0.0–17.3)
Arousal index, events/h	17.8 ± 9.5 (0.5–39.4)
ODI, events/h	4.9 ± 4.1 (0.0–15.5)
ODI REM, events/h	12.2 ± 12.2 (0.0–41.5)
ODI NREM, events/h	3.3 ± 3.4 (0.0–15.7)
Mean SpO_2_, %	91.8 ± 2.5 (84.4–95.7)
Minimum SpO_2_, %	86.3 ± 4.2 (77.0–92.0)
SpO_2_ < 90%, % TST	15.3 ± 29.2 (0.0–99.9)
SpO_2_ < 90%, min	47.4 ± 91.6 (0.0–321.3)
Snoring, % TST	11.1 ± 20.3 (0.0–80.5)
PLMs, events/h	20.0 ± 38.8 (0.0–185.1)
ESS score	6.5 ± 3.8 (0–22)
ESS score > 10, n (%)	6 (14)

Values are expressed as mean ± standard deviation (range) or as number of patients (percentage). Abbreviations: AHI, apnea–hypopnea index; ESS, Epworth Sleepiness Scale; NREM, non-rapid eye movement sleep; ODI, oxygen desaturation index; PLMs, periodic limb movements; REM, rapid eye movement sleep; SpO_2_, peripheral oxygen saturation; TST, total sleep time.

**Table 3:** Clinical characteristics and polysomnographic findings in adult people with cystic fibrosis (pwCF) with and without obstructive sleep apnea (OSA).

Parameter	OSA (n = 21)	No OSA (n = 23)	*p*-Value
Age, years	33.4 ± 9.7	29.1 ± 6.7	0.171
Male/Female, n	16/5	14/9	0.276
BMI, kg/m^2^	22.0 ± 3.7	21.2 ± 3.4	0.708
Lund–Mackay score (LMS)	13.8 ± 5.3	15.2 ± 5.0	0.355
Main nasal cavity score (MNCS)	1.0 ± 0.7	1.0 ± 0.6	0.944
LMS + MNCS	14.7 ± 5.7	16.2 ± 5.3	0.383
TST, min	302.7 ± 27.2	305.0 ± 46.4	0.846
Sleep efficiency, %	76.7 ± 8.4	75.4 ± 10.3	0.645
AHI, events/h	9.0 ± 3.4	1.9 ± 1.3	<0.001
AHI REM, events/h	20.8 ± 13.3	5.3 ± 5.0	<0.001
AHI NREM, events/h	6.3 ± 3.8	1.2 ± 1.0	<0.001
Arousal index, events/h	21.2 ± 8.8	14.7 ± 9.1	0.021
ODI, events/h	8.4 ± 3.3	1.8 ± 1.2	<0.001
ODI REM, events/h	20.4 ± 12.7	5.1 ± 5.0	<0.001
ODI NREM, events/h	5.7 ± 3.5	1.2 ± 1.0	<0.001
Mean SpO_2_, %	90.6 ± 2.9	92.9 ± 1.6	0.002
Minimum SpO_2_, %	83.1 ± 3.4	89.2 ± 2.1	<0.001
SpO_2_ < 90%, % TST	30.2 ± 37.0	1.7 ± 4.4	<0.001
SpO_2_ < 90%, min	94.2 ± 116.2	4.7 ± 11.4	<0.001
Snoring, % TST	15.0 ± 21.3	7.5 ± 19.2	0.049
PLMI, events/h	32.8 ± 52.0	8.6 ± 13.7	0.013
ESS score	6.7 ± 4.3	6.3 ± 3.5	0.935
FEV_1_, L	2.0 ± 0.7	2.2 ± 0.9	0.316
FEV_1_, % predicted	47.9 ± 14.6	55.4 ± 16.1	0.111
FVC, L	3.2 ± 1.2	3.5 ± 1.1	0.446
FVC, % predicted	64.3 ± 19.3	73.6 ± 16.4	0.093
FEF_25-75_, L/s	0.82 ± 0.5	1.2 ± 0.9	0.117
FEF_25-75_, % predicted	19.8 ± 11.8	27.0 ± 16.4	0.107

Values are presented as mean ± standard deviation, unless stated otherwise. AHI, apnea–hypopnea index; BMI, body mass index; ESS, Epworth Sleepiness Scale; FEF_25-75_, forced expiratory flow between 25% and 75% of vital capacity; FEV_1_, forced expiratory volume in one second; FVC, forced vital capacity; NREM, non-rapid eye movement sleep; ODI, oxygen desaturation index; PLMI, periodic limb movement index; REM, rapid eye movement sleep; SpO_2_, peripheral oxygen saturation; TST, total sleep time.

**Table 4:** Correlations between sinus computed tomography (CT)–derived scores and polysomnographic parameters or Epworth Sleepiness Scale (ESS) score in adult people with cystic fibrosis (pwCF).

Sinus-CT Score		AHI, Events/h	AHI REM, Events/h	AHI NREM, Events/h	Snoring, % TST	Arousal Index, Events/h	ESS Score
LMS	r	−0.14	−0.25	−0.03	0.03	−0.03	0
	*p*-value	0.356	0.095	0.871	0.824	0.857	0.993
MNCS	r	0.02	0.08	0.01	0.14	0.22	−0.02
	*p*-value	0.875	0.592	0.934	0.382	0.15	0.893
LMS + MNCS	r	−0.13	−0.23	−0.02	0.05	0	0
	*p*-value	0.396	0.133	0.886	0.759	0.991	0.994

AHI, apnea–hypopnea index; ESS, Epworth Sleepiness Scale; LMS, Lund–Mackay Score; MNCS, Main Nasal Cavity Score; NREM, non-rapid eye movement sleep; REM, rapid eye movement sleep; r, Pearson correlation coefficient.

**Table 5:** Clinical characteristics and polysomnographic findings in adult people with cystic fibrosis (pwCF) in relation to treatment with CFTR modulators (tezacaftor/ivacaftor or lumacaftor/ivacaftor).

	CFTR Modulator Use		
Parameter	Yes (n = 31)	No (n = 13)	*p*-Value
Age, years	31.1 ± 8.6	30.2 ± 8.4	0.650
Male/Female, n	20/11	10/3	0.420
BMI, kg/m^2^	21.5 ± 3.6	21.9 ± 3.4	0.731
Lund–Mackay score (LMS)	14.3 ± 4.9	15.2 ± 5.8	0.617
Main nasal cavity score (MNCS)	0.9 ± 0.6	1.0 ± 0.6	0.752
LMS + MNCS	15.2 ± 5.3	16.2 ± 6.1	0.613
TST, min	310.3 ± 34.6	288.8 ± 42.9	0.088
Sleep efficiency, %	77.4 ± 8.5	72.6 ± 10.6	0.175
AHI, events/h	5.9 ± 4.9	3.9 ± 2.4	0.458
AHI REM, events/h	13.2 ± 13.4	11.4 ± 10.4	0.969
AHI NREM, events/h	4.2 ± 4.2	2.4 ± 1.8	0.481
Arousal index, events/h	18.3 ± 9.9	16.5 ± 8.4	0.581
ODI, events/h	5.5 ± 4.6	3.7 ± 2.3	0.481
ODI REM, events/h	15.2 ± 17.6	11.3 ± 10.6	0.739
ODI NREM, events/h	3.8 ± 3.8	2.4 ± 1.6	0.663
Mean SpO_2_, %	91.6 ± 2.8	92.1 ± 1.9	0.581
Minimum SpO_2_, %	86.2 ± 4.5	86.7 ± 3.3	0.918
SpO_2_ < 90%, % TST	17.3 ± 31.0	11.8 ± 26.4	0.772
SpO_2_ < 90%, min	53.4 ± 95.3	37.3 ± 87.8	0.895
Snoring, % TST	11.8 ± 21.3	9.4 ± 18.4	0.573
PLMI, events/h	24.1 ± 45.2	10.1 ± 12.4	0.489
ESS score	6.6 ± 4.0	6.1 ± 3.4	0.522
FEV_1_, L	1.9 ± 0.7	2.5 ± 1.0	0.096
FEV_1_, % predicted	48.7 ± 13.8	59.2 ± 18.1	0.043
FVC, L	3.2 ± 1.1	3.7 ± 1.3	0.288
FVC, % predicted	67.1 ± 17.5	74.1 ± 19.9	0.276
FEF_25-75_, L/s	0.87 ± 0.6	1.31 ± 1.0	0.089
FEF_25-75_, % predicted	21.0 ± 12.9	29.6 ± 17.2	0.082

Values are presented as mean ± standard deviation, unless stated otherwise. AHI, apnea–hypopnea index; BMI, body mass index; CFTR, Cystic Fibrosis transmembrane conductance regulator; ESS, Epworth Sleepiness Scale; FEF_25-75_, forced expiratory flow between 25% and 75% of vital capacity; FEV_1_, forced expiratory volume in one second; FVC, forced vital capacity; NREM, non-rapid eye movement sleep; ODI, oxygen desaturation index; PLMI, periodic limb movement index; REM, rapid eye movement sleep; SpO_2_, peripheral oxygen saturation; TST, total sleep time.

## Data Availability

The original contributions presented in this study are included in the article. Further inquiries can be directed at the corresponding author.
